# Early prediction of immune checkpoint inhibitor-related pneumonitis in advanced non-small cell lung cancer based on primary tumor delta-radiomics features

**DOI:** 10.3389/fimmu.2026.1795169

**Published:** 2026-05-28

**Authors:** Dong Xie, Lingang Xu, Jinxia Xu, Haifeng Chen, Jinna Yu, Cong He, Yonggang Qiu, Linfeng Fu, Qiu Han, Lingting Kong, Fangye Wu

**Affiliations:** 1 Department of Radiology, Shaoxing Second Hospital, Shaoxing, China; 2 Department of Interventional Radiology Suite, Shaoxing Second Hospital, Shaoxing, Zhejiang, China; 3 Department of Medical Oncology, Shaoxing Second Hospital, Shaoxing, China

**Keywords:** delta, immunity, machine learning, non-small cell lung cancer, prediction model, radiomics

## Abstract

**Objective:**

To investigate the effectiveness of predicting immune checkpoint inhibitor-related pneumonitis (ICIP) in patients with advanced non-small cell lung cancer (NSCLC) using Delta radiomics features derived from pre- and post-treatment enhanced CT images.

**Methods:**

This single-center retrospective study extracted radiomics features of primary tumors from baseline enhanced CT images and from enhanced CT images obtained after the first to third treatment cycles in patients with stage IIIB-IV NSCLC receiving immune checkpoint inhibitors (ICIs). Differences between features were calculated as Delta features. Feature selection was performed using the Least Absolute Shrinkage and Selection Operator (LASSO) regression. Prediction models were developed using Logistic Regression (LR), Support Vector Machine (SVM), K-Nearest Neighbors (KNN), and Extreme Gradient Boosting (XGBoost) algorithms. These models were compared and further integrated with a clinical feature-based model incorporating a history of interstitial lung disease, absolute lymphocyte count, and neutrophil/lymphocyte ratio. Model performance was assessed using five-fold cross-validation.

**Results:**

A total of 131 patients were included, among whom 46 (35.1%) developed ICIP, including 8 patients (17.4%) with grade 3–5 ICIP. From 2153 initial features, 22 key Delta radiomics features were selected for model construction. The Delta radiomics model based on the LR algorithm showed the best performance in both the training and validation sets, with AUCs of 0.92 (95% CI: 0.88-0.97) and 0.85 (95% CI: 0.78-0.92), respectively. After integration with the clinical model, the performance of the combined model was further improved in the training set, achieving an AUC of 0.94 (95% CI: 0.90-0.98), while the validation set AUC was 0.86 (95% CI: 0.79–0.93). Although the difference in AUC between the combined model and the LR model in the validation set was not statistically significant (DeLong test, P = 0.4691). Calibration curves and decision curve analysis indicated good calibration and favorable clinical utility.

**Conclusion:**

This preliminary model offers a potential imaging-based biomarker for early risk stratification of any-grade ICIP in patients with advanced NSCLC. Performance specifically for high-grade (grade 3-5) ICIP could not be evaluated due to the limited number of such events. External validation in independent cohorts is required before clinical application.

## 1 Introduction

Non-small cell lung cancer (NSCLC) accounts for approximately 85% of lung cancers and has become a leading cause of cancer-related mortality worldwide (1, 2). Squamous cell carcinoma and adenocarcinoma are its main histological types (3). The treatment paradigm for advanced NSCLC has undergone a fundamental transformation, with outstanding efficacy supported by recent pivotal clinical studies and guidelines. Immune checkpoint inhibitors (ICIs) are now the core first-line treatment for advanced NSCLC without driver gene mutations. They have significantly improved overall survival and shifted treatment goals toward long-term disease control (4–7). During the tumor immune response, T cells serve as the primary effector cells. Upon activation, they accumulate in large numbers and attack tumor cells expressing relevant antigens. Immune checkpoints such as Cytotoxic T-Lymphocyte-Associated Antigen 4 (CTLA-4), Programmed Cell Death Protein 1 (PD-1), and PD-L1 can inhibit T cell initiation and activation, allowing tumor cells to evade immune surveillance (8, 9). Currently, the ICIs used for NSCLC mainly include Nivolumab, Pembrolizumab, Cemiplimab, Atezolizumab, Avelumab, Durvalumab, and Ipilimumab (10). However, while immunotherapy improves patient survival, it can also trigger a range of immune-related adverse events (IRAEs), affecting the skin, respiratory system, gastrointestinal system, and cardiovascular system (11–13).

Immune checkpoint inhibitor-related pneumonitis (ICIP) is a potentially life-threatening inflammatory lung disease caused by ICIs therapy. It is one of the most common severe IRAEs aside from skin toxicities (14, 15). The exact mechanism is not fully understood, but it is generally believed to be related to abnormal immune activation following the release of T cell inhibition by ICIs. This process leads to immune-mediated attacks on normal lung tissues (16). ICIP is a significant cause of treatment interruption and even death in patients receiving ICIs, which requires clinicians to pay close attention to early recognition and timely intervention. Its clinical manifestations are diverse and range from asymptomatic imaging abnormalities to rapidly progressive respiratory failure. Common symptoms include dyspnea, which is the most frequent manifestation, dry cough, fever, and chest pain. Some patients may remain completely asymptomatic, with abnormalities detected only during imaging follow-up (17, 18). The imaging features of ICIP are diverse and non-specific. High-resolution CT is the preferred imaging modality for the diagnosis and evaluation of ICIP. Typical findings include ground-glass opacities and/or consolidations distributed in both lungs, which may appear diffuse, patchy, or peripheral. In some cases, bronchovascular bundle thickening, reticular patterns, or interlobular septal thickening may also be observed (19). Currently, there is no established gold standard for the diagnosis of ICIP. Clinicians and radiologists usually rely on subjective interpretation based on patient symptoms and imaging findings (18). Diagnosis mainly involves a comprehensive assessment of clinical manifestations, imaging examinations, and laboratory tests. Patients presenting with acute dyspnea, cough, fever, or other pneumonia-like symptoms after ICIs therapy should first have other pulmonary diseases excluded. Imaging studies, particularly chest CT, often show diffuse or localized ground-glass opacities, pulmonary consolidation, or features of interstitial lung disease. Laboratory examinations, including complete blood count, liver and kidney function tests, and pulmonary function tests, together with microbiological investigations such as sputum culture and bronchoalveolar lavage fluid analysis, can help exclude infections and other potential causes.

Radiomics is an emerging interdisciplinary field that focuses on the high-throughput extraction of a large number of quantitative features from standard medical imaging, such as CT, MRI, and PET, and on transforming these data into mineable imaging characteristics (20). By integrating machine learning (ML) algorithms, radiomics aims to construct models for diagnosis, prognostic prediction, and therapeutic efficacy evaluation, thereby supporting clinical decision-making and promoting the development of precision medicine. It plays an increasingly important role in the assessment of tumor heterogeneity and in precision diagnosis and treatment. Promising results have been achieved in several areas, including the differentiation of benign and malignant pulmonary nodules and automatic segmentation (21), non-invasive prediction of pathological types and genetic phenotypes (22), efficacy prediction and evaluation, particularly for immunotherapy (23), and prognostic prediction and risk stratification (24). Delta-radiomics represents an important frontier branch of radiomics. It is not limited to the analysis of static images at a single time point but instead focuses on dynamic changes in radiomic features before and after treatment, such as radiotherapy, chemotherapy, or immunotherapy. This approach enables a more accurate assessment of changes in tumor heterogeneity and treatment response (25). Efficacy evaluation and early response prediction are the core applications of Delta-radiomics. By comparing imaging feature changes before treatment and at an early treatment stage, Delta-radiomics can identify responders and non-responders more rapidly and sensitively than traditional tumor size measurements based on the Response Evaluation Criteria in Solid Tumors (RECIST). Fave X et al. (26) demonstrated that Delta-radiomic features extracted from pre- and post-radiotherapy CT images significantly outperformed models using only pre-treatment features in predicting overall survival in patients with NSCLC, thereby establishing the prognostic value of Delta-radiomics. Mu W et al. (27) developed a “treatment response score” based on deep feature changes derived from pre- and post-treatment PET/CT images to predict whether patients with NSCLC receiving neoadjuvant immunotherapy achieved major pathological remission.

This study focuses on patients with advanced NSCLC and aims to analyze changes in enhanced CT images obtained before and after treatment to extract Delta radiomic features that reflect tumor treatment response. On this basis, we seek to construct a Delta radiomic model that integrates treatment-related change information and to explore whether this model can predict ICIP induced by immune checkpoint inhibitors (ICIs) at an early stage. The model is also intended to assist in identifying patients who are most likely to benefit from immunotherapy while having a lower risk of ICIP. Of note, even low-grade ICIP warrants clinical attention due to potential treatment disruption and progression, so any-grade prediction is useful as an early warning signal, but our study does not specifically predict high-grade events. Ultimately, if validated in future multi-center studies, this approach may contribute to personalized immunotherapy decision-making. However, the present study is exploratory and aimed at generating hypotheses for further validation.

## 2 Materials and methods

### 2.1 Patients

A retrospective analysis was conducted on patients with advanced NSCLC who received ICIs treatment at our hospital from January 2018 to March 2025. Tumor staging was performed according to the 8th edition of the American Joint Committee on Cancer TNM staging system (28), and all patients were pathologically confirmed as having stage IIIB to IV NSCLC. This study complied with the principles of the Declaration of Helsinki and was approved by the hospital ethics committee, with the Approval No.: Shaoxing Second Hospital Ethics Committee Review 2024 Research Serial No. 066.

Inclusion criteria were as follows: (1) histologically confirmed NSCLC; (2) first-line treatment with ICIs; (3) complete baseline demographic data available before treatment. Exclusion criteria were as follows: (1) no comparative enhanced CT examination performed after baseline imaging or after 1 to 3 treatment cycles; (2) enhanced CT images that could not accurately evaluate lesion boundaries; (3) a history of prior chest surgery or prior thoracic radiotherapy; (4) presence of extra-pulmonary primary tumors.

### 2.2 CT examination method

A 64-slice CT scanner (Siemens SOMATOM Definition AS, Germany) was used for all examinations, and routine breathing training was performed before scanning. The scanning parameters were as follows: tube voltage 120 kV, tube current 200–300 mA, rotation time 0.75 seconds, detector collimation 32x1.25 mm, field of view (FOV) 36.00–50.00 cm, matrix size 512x512, slice thickness and interval both set to 5.0 mm, contrast agent injection rate 2.5–3.0 ml/s, and injection volume 1.1–1.7 ml/kg. After the routine scan, thin-slice reconstruction was performed with a slice thickness of 0.6–1.5 mm.

### 2.3 Image analysis

ICIP was defined as newly appearing pulmonary opacities after immunotherapy, with other pulmonary infections or tumor progression excluded. All ICIP events were graded according to the Common Terminology Criteria for Adverse Events (CTCAE) version 5.0. ICIP diagnosis and grade assignment were performed jointly by two independent reviewers: a radiologist (with 17 years of experience in thoracic imaging) and a medical oncologist (with 14 years of experience in immunotherapy). In cases of disagreement, a consensus was reached by discussion. Differential diagnoses included pulmonary infections such as bacterial, viral, fungal, and mycoplasma or chlamydia infections, tumor progression or pseudoprogression, radiation pneumonitis, pulmonary embolism, and cardiogenic pulmonary edema. No pathological confirmation of ICIP was obtained, since non-invasive diagnosis based on clinical and imaging criteria is standard practice in this context.

### 2.4 Tumor segmentation

In this study, the Deepwise Medical Multi-modal Research Platform version 3.0.0 (https://keyan.deepwise.com, Beijing Deepwise & League of PHD Technology Co., Ltd, Beijing, China) was used for data analysis. This platform is an integrated machine learning system for medical data analysis and incorporates mature Python libraries, including pyradiomics (version 3.0.1) and scikit-learn (version 0.22). Fully automated segmentation technology was applied to delineate the regions of interest (ROI) of lesions slice by slice on contrast-enhanced CT images obtained at baseline (time point 0, TP0) and after the 1st to 3rd cycles of immunotherapy (time point 1, TP1). After automated segmentation, each ROI was visually inspected by a single radiologist (with 13 years of experience in thoracic imaging). Manual corrections were applied when the automated segmentation was clearly inaccurate (e.g., inclusion of adjacent vessels, chest wall, or atelectasis, or incomplete tumor coverage). The final ROIs were then approved by a second senior radiologist (with 17 years of experience).

### 2.5 Feature extraction

For each three-dimensional region of interest (ROI), seven categories of features were extracted, including first-order features, shape features, gray level co-occurrence matrix (GLCM), gray level run length matrix (GLRLM), gray level size zone matrix (GLSZM), gray level dependence matrix (GLDM), and neighborhood gray-tone difference matrix (NGTDM). Delta features (Delta-RFs) were defined as the net changes in features between TP0 and TP1, calculated as Delta-RFs = Feature (TP1) – Feature (TP0).

### 2.6 Feature selection

A five-fold cross-validation (CV) strategy was applied for feature selection and model evaluation. The entire dataset was first stratified by ICIP status and randomly divided into 5 folds using a fixed random seed. In each fold, 80% of the data served as the training set and the remaining 20% as the validation set. The fold indices were fixed before any analysis.

To avoid data leakage, all preprocessing and feature selection steps were performed independently within each CV fold, using only the training set of that fold. The steps were as follows: (1) Normalization: Z-score normalization was performed using the mean and standard deviation calculated from the training set of the current fold; the same parameters were then applied to normalize the corresponding validation set. (2) Correlation filtering: Pairwise Pearson correlation coefficients were calculated on the normalized training set. Features with a correlation coefficient >0.9 were removed as redundant. The same feature indices were then excluded from the validation set of the same fold. (3) LASSO feature selection: Using only the normalized and filtered training set, LASSO regression with nested five-fold cross-validation was used to determine the optimal regularization parameter λ (the value minimizing the cross-validation deviance). Features with non-zero coefficients were selected. Only the corresponding features were retained in the validation set.

The above procedure was repeated for each of the 5 folds. To obtain a stable set of features for final model construction, we retained features that were selected in at least 4 out of 5 folds. After feature selection, the final model performance was evaluated on the validation sets of each fold, and the reported metrics represent the average across the five folds.

### 2.7 Construction of radiomics and clinical prediction models

Four ML algorithms were applied in this study to predict ICIP based on the selected features, including Logistic Regression (LR), linear support vector machine (SVM), k-Nearest Neighbor (KNN), and eXtreme Gradient Boosting (XGBoost). The machine learning models were implemented using the default parameters provided by the Deepwise platform (based on scikit-learn and XGBoost libraries). The hyperparameter settings were as follows:

- Logistic Regression: C = 1, penalty = ‘l2’, fit_intercept = False, class_weight = ‘balanced’.*
- Support Vector Machine (SVM): kernel = ‘sigmoid’, C = 1, gamma = ‘auto’, degree = 3, coef0 = 0, tol = 1e-4, shrinking = True, class_weight = ‘balanced’.*
- K-Nearest Neighbors (KNN): n_neighbors = 5, weights = ‘uniform’, p = 2 (Euclidean distance).*
- XGBoost: booster = ‘gblinear’ (linear booster). *

In addition, a clinical prediction model was constructed using LR based on the selected clinical features. By integrating the optimal ML model with the clinical model, we aimed to improve predictive accuracy, reduce overfitting, and enhance the stability and generalizability of the prediction model.

### 2.8 Model evaluation

**Figure 1:**
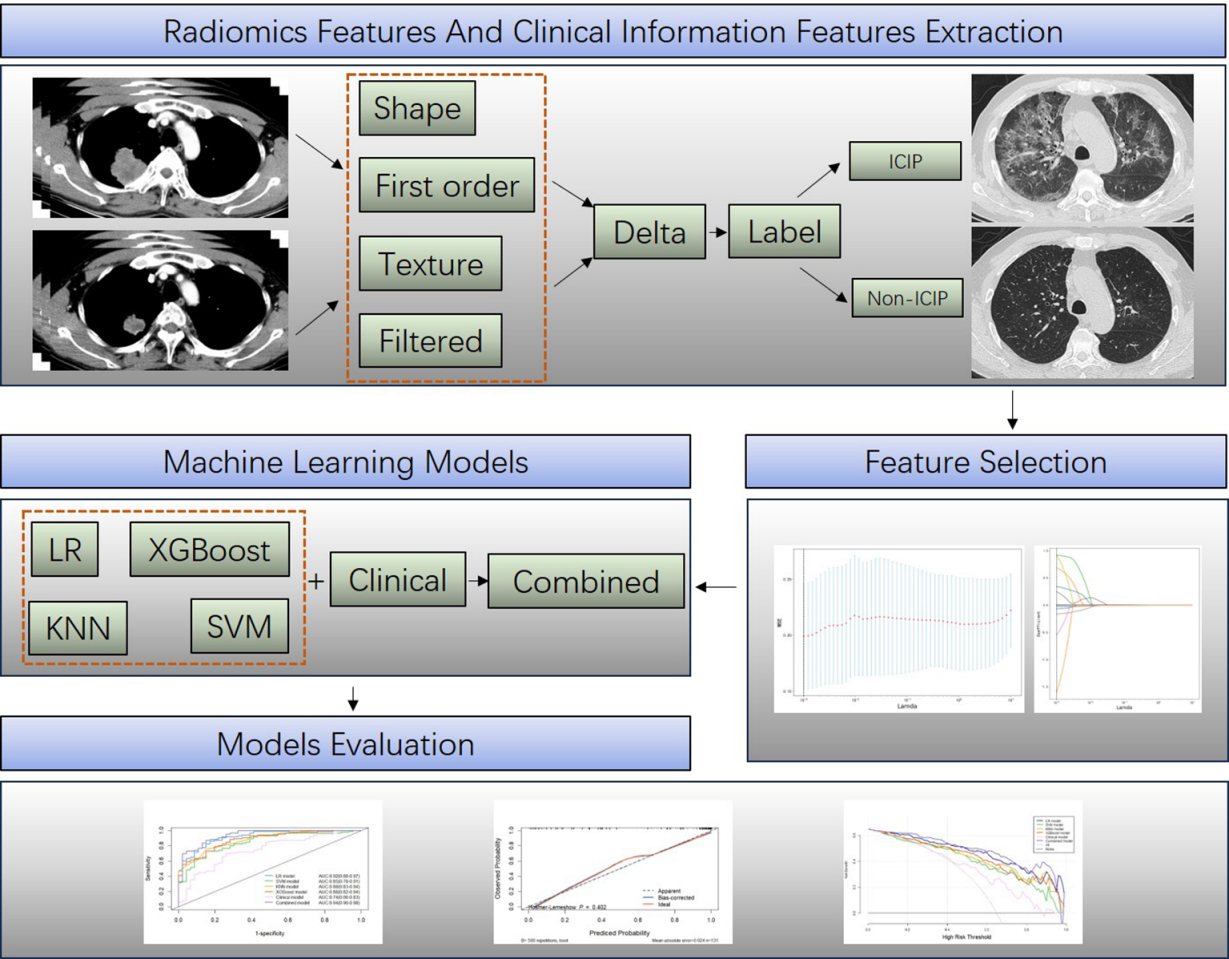
Workflow of this study, which is mainly composed of six steps: data set, feature extraction, feature selection, model building, analysis, and validation.

Model predictive performance was assessed using the area under the receiver operating characteristic (ROC) curve, expressed as AUC. The DeLong test was used to compare performance differences among models. Calibration curves were generated to qualitatively evaluate model calibration. Decision curve analysis (DCA) was performed to assess the clinical utility of the prediction models by estimating net benefits across different threshold probabilities. In addition, multiple performance indicators were calculated for each model to quantitatively evaluate predictive ability. All models were validated using the validation set. The overall workflow of this study is presented in **Figure 1**.

### 2.9 Statistical methods

The Deepwise Multimodal Research Platform version 3.0.0 (https://keyan.deepwise.com) was used as the primary statistical analysis tool. SPSS software (version 26.0) and R software (version 4.1.2, https://www.r-project.org/) were also used for data analysis. Continuous variables with a normal distribution are presented as mean ± standard deviation (X^-^ ± S), and the independent samples t-test was applied. Non-normally distributed data are expressed as median (interquartile range) [M(Q1, Q3)] and were analyzed using the Wilcoxon rank-sum test. Categorical variables are presented as percentages [n(%)] and were analyzed using the Chi-square (χ^2^) test. A P value < 0.05 was considered statistically significant.

## 3 Results

### 3.1 Demographic information

A total of 312 patients with stage IIIB-IV non-small cell lung cancer who received immunotherapy between January 2018 and March 2025 were initially collected. According to the exclusion criteria, 181 cases were excluded. Finally, 131 patients with NSCLC were included in this study, comprising 112 males (85.5%) and 19 females (14.5%). The patients ranged in age from 49 to 90 years, with a mean age of (69.82 ± 5) years. Among these patients, 46 cases (35.1%) were classified into the ICIP group and 85 cases (64.9%) into the Non-ICIP group. Among the 46 ICIP events, severity grades based on the CTCAE version 5.0 were as follows: grade 1 in 21 patients, grade 2 in 17 patients, grade 3 in 7 patients, grade 4 in 1 patient, and no grade 5 events. The median interval between TP0 and the start of immunotherapy was 11 days (range: 0–31 days). The median interval between TP0 and TP1 was 49 days (range: 18–85 days). Comparisons of demographic and clinicopathologic characteristics between the ICIP and Non-ICIP groups are presented in **Table 1**. Statistically significant differences were observed in “Interstitial lung diseases (ILDs),” “Absolute Lymphocyte Count (ALC),” and “Neutrophil-to-Lymphocyte Ratio (NLR)” between the two groups (P < 0.05).

**Table 1:** Baseline data of the ICIP and non-ICIP cohorts.

Demographic or clinicopathologic characteristic	Non-ICIP	ICIP	P
	(N=85)	(N=46)	
Gender, No. (%)			0.727
Female	13 (15.3%)	6 (13.0%)	
Male	72 (84.7%)	40 (87.0%)	
Age, (mean SD)	68.529 ± 9.300	67.109 ± 9.909	0.416
Tobacco use, No. (%)			0.401
smoker	49 (57.6%)	23 (50.0%)	
Non-smoker	36 (42.4%)	23 (50.0%)	
Pathological type, No. (%)			0.103
Squamous cell	65 (76.5%)	29 (63.0%)	
Adenocarcinoma	20 (23.5%)	17 (37.0%)	
Anatomical classification, No. (%)			0.548
Central type	49 (57.6%)	29 (63.0%)	
Peripheral type	36 (42.4%)	17 (37.0%)	
TNM Stage, No. (%)			0.413
III	30 (35.3%)	13 (28.3%)	
IV	55 (64.7%)	33 (71.7%)	
Immunotherapy concurrent with chemotherapy			0.336
No	21 (24.7%)	8 (17.4%)	
Yes	64 (75.3%)	38 (82.6%)	
Immunotherapy concurrent with targeted therapy			0.503
No	66 (77.6%)	38 (82.6%)	
Yes	19 (22.4%)	8 (17.4%)	
ILDs			0.030 *
No	74 (87.1%)	33 (71.7%)	
Yes	11 (12.9%)	13 (28.3%)	
Emphysema			0.695
No	62 (72.9%)	35 (76.1%)	
Yes	23 (27.1%)	11 (23.9%)	
Pleural effusion			0.969
No	57 (67.1%)	31 (67.4%)	
Yes	28 (32.9%)	15 (32.6%)	
ALC (x 10^9^/l)	1.380(0.945-1.650)	1.090(0.890-1.350)	0.017 *
NLR	4.180 ± 2.394	5.927 ± 3.787	0.002 *

ILDs, interstitial lung diseases; ALC, absolute lymphocyte count; NLR, neutrophil/lymphocyte ratio, * Significant difference (P < 0.05).

### 3.2 Model building

**Figure 2:**
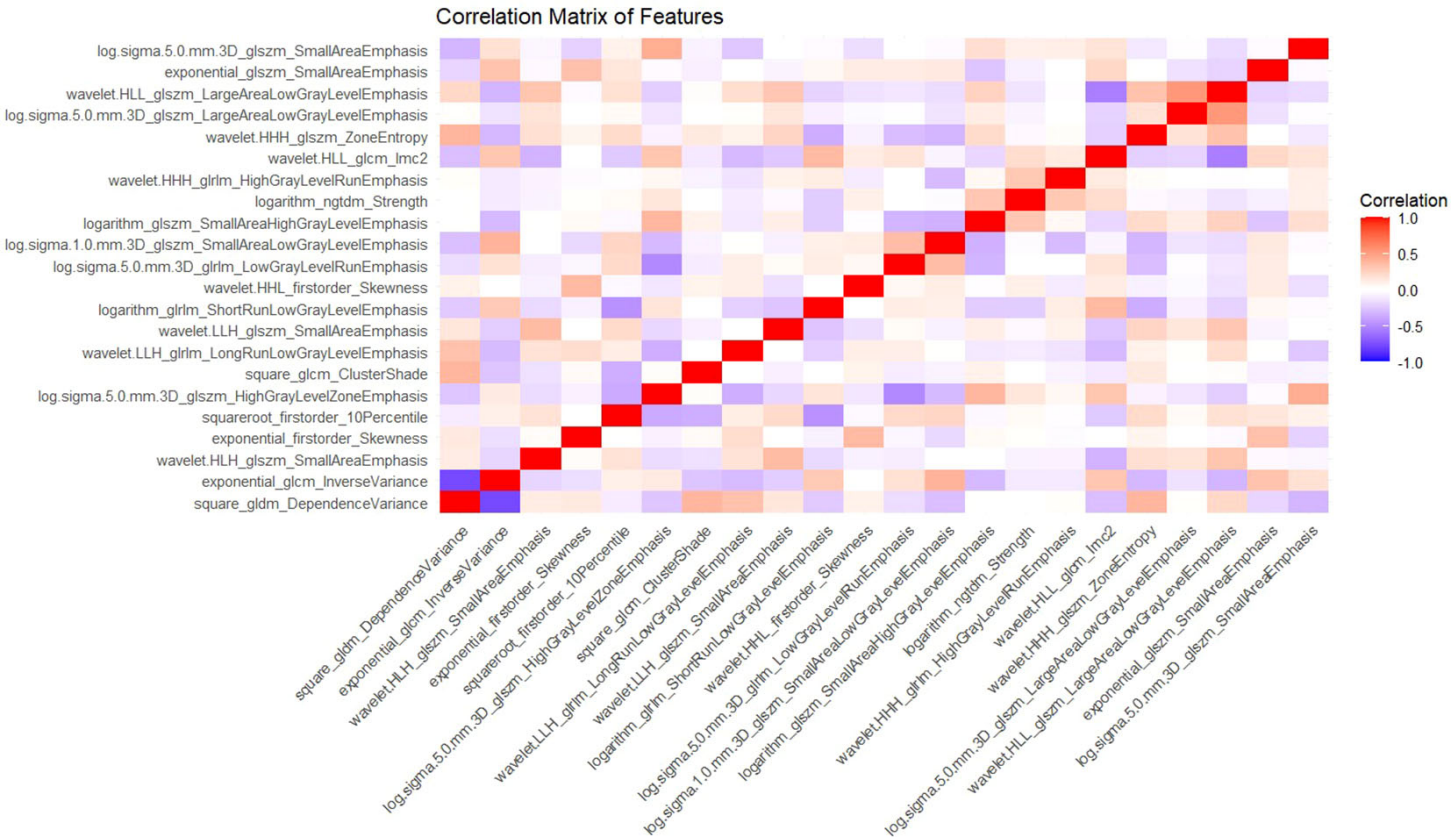
Heatmap of radiomics feature correlations. This heatmap visualizes the correlation matrix among the selected radiomics features.

A total of 2153 radiomics features were extracted, and the LASSO method was applied to remove irrelevant or redundant features. Ultimately, 22 features were selected for the construction of the machine learning models, and the Pearson correlation analysis heatmap of these features is shown in **Figure 2**. Among the 22 selected Delta radiomics features, most were texture-based features. GLSZM features accounted for 10 (45.5%), followed by GLRLM features (4, 18.2%), GLCM features (3, 13.6%), and first-order statistics (3, 13.6%). The remaining features included one GLDM and one NGTDM feature. No shape features were retained. In terms of image filters, wavelet-filtered features were the most common (8, 36.4%), followed by Laplacian of Gaussian (LoG)-filtered features (5, 22.7%), exponential-transformed features (3, 13.6%), logarithm-transformed features (3, 13.6%), square-transformed features (2, 9.1%), and square root-transformed features (1, 4.5%). The predominance of wavelet and LoG filtering suggests that the model captures textural heterogeneity at different scales. The correlation heatmap (**Figure 2**) shows low inter-feature correlations, which indicates limited redundancy after LASSO selection. The full list of selected features, along with their filters and families, is provided in **Supplementary Table 1**.

To assess whether the timing of the follow-up CT scan influenced the magnitude of the Delta-radiomics features, we calculated Spearman correlations between the top five features (by absolute LASSO coefficient) and the TP0-TP1 interval. As shown in **Supplementary Table 2**, none of the correlations reached statistical significance (all P > 0.05), with ρ values ranging from –0.133 to 0.087. This suggests that, within the observed time window (median 49 days; range 18–85 days), the Delta-radiomics feature values were not systematically driven by the timing of the scan.

Feature importance was determined using LASSO regression. LASSO was applied uniformly across all machine learning models to ensure a model-agnostic feature selection strategy, which allowed consistent feature input and fair performance comparison among different algorithms. Based on the selected features, the LR, SVM, KNN, and XGBoost algorithms were used to train delta radiomics features for each primary tumor, resulting in the LR model, SVM model, KNN model, and XGBoost model. The output probability scores of these models were transformed into delta radiomics scores. Significant differences in the distribution of delta radiomics scores were observed between the ICIP and Non-ICIP groups (**Supplementary Figure 1**). Using the LR method, three key clinical features were ultimately selected to construct the Clinical model, namely “ILDs,” “ALC,” and “NLR.”.

### 3.3 Model performance

**Figure 3:**
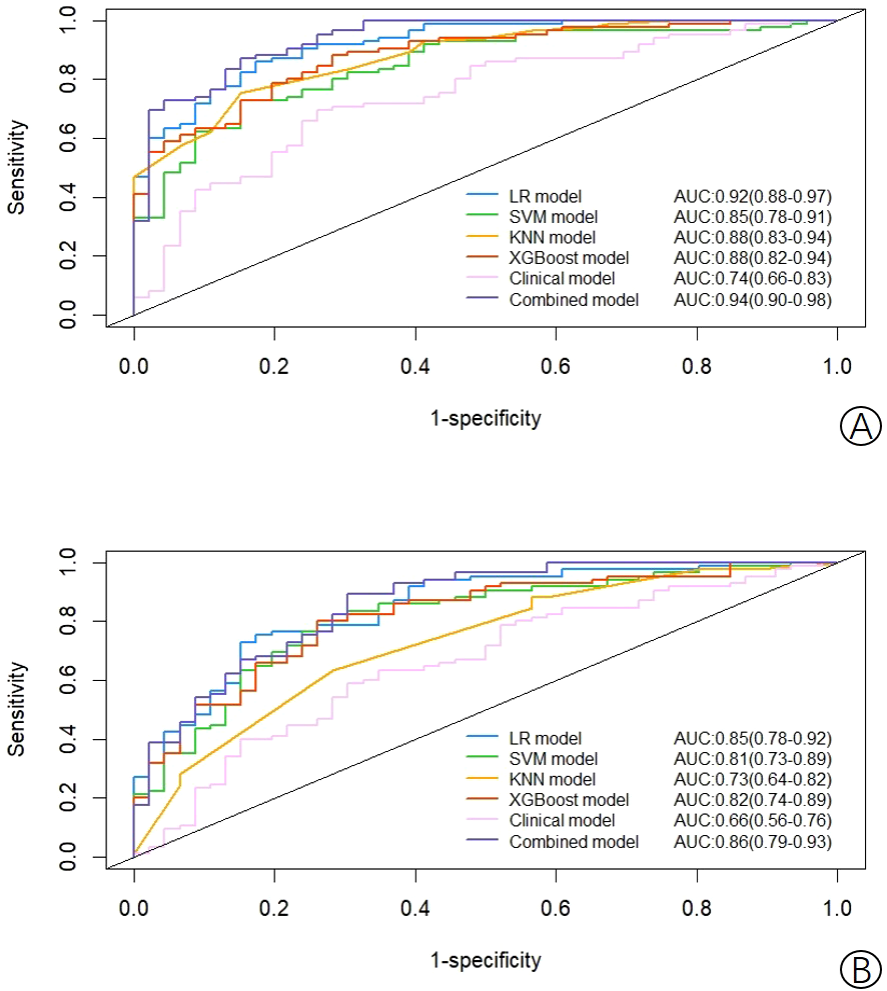
AUC values of the LR model, SVM model, KNN model, XGBoost model, clinical model, and combined model in the training cohort **(A)** and validation cohort **(B)**.

In both the training and validation sets, the LR model achieved higher AUC values than the other machine learning models and the Clinical model, with AUCs of 0.92 (95% CI = 0.88–0.97) and 0.85 (95% CI = 0.78–0.92), respectively. When the LR model was combined with the Clinical model, the AUC of the Combined model exceeded that of the LR model alone, increasing from 0.92 to 0.94 in the training set and from 0.85 to 0.86 in the validation set. The DeLong test showed no statistically significant difference between the two models (p = 0.4691). Thus, the incremental predictive benefit of adding clinical features to the LR model was not confirmed in the validation cohort. In the training set, the AUC of the Combined model (0.94, 95% CI = 0.90–0.98) was significantly higher than that of the SVM model (0.85, 95% CI = 0.78–0.91), the KNN model (0.88, 95% CI = 0.83–0.94), the XGBoost model (0.88, 95% CI = 0.82–0.94), and the Clinical model (0.74, 95% CI = 0.66–0.83), as confirmed by the DeLong test (all P < 0.05). In the validation set, the AUC of the Combined model (0.86, 95% CI = 0.79–0.93) was significantly higher than that of the KNN model (0.73, 95% CI = 0.64–0.82) and the Clinical model (0.66, 95% CI = 0.56–0.76) (DeLong test, P < 0.05) (**Figure 3**, **Table 2**).

**Table 2:** DeLong tests for comparison of AUCs between the combined model and machine learning models.

Comparison	Training cohort		Validation cohort	
	AUC	P value	AUC	P value
Combined model vs LR model	0.94	0.1480	0.86	0.4691
	0.92		0.85	
Combined model vs SVM model	0.94	0.0003**	0.86	0.0748
	0.85		0.81	
Combined model vs KNN model	0.94	0.0277*	0.86	0.0002**
	0.88		0.73	
Combined model vs XGBoost model	0.94	0.0108*	0.86	0.1032
	0.88		0.82	
Combined model vs Clinical model	0.94	< 0.0001**	0.86	< 0.0001**
	0.74		0.66	

LR, logistic regression; SVM, linear support vector machine; KNN, k-Nearest Neighbor; XGBoost, eXtreme Gradient Boosting; * Significant difference (P < 0.05); ** Extremely significant difference (P < 0.001).

The performance of the Combined model was further compared with that of the individual machine learning models and the Clinical model. **Table 3** summarizes the commonly used evaluation metrics. Across the LR, SVM, KNN, XGBoost, Clinical, and Combined models, the Combined model achieved the highest AUC in both the training cohort (0.94) and the validation cohort (0.86), indicating strong discriminative ability and good generalization performance. In the training cohort, the Combined model achieved an AUC of 0.94, with an accuracy of 0.85, precision of 0.91, recall of 0.85, F1 score of 0.88, sensitivity of 0.85, specificity of 0.85, positive predictive value (PPV) of 0.91, and negative predictive value (NPV) of 0.75. In the validation cohort, the AUC was 0.86, with an accuracy of 0.75, precision of 0.84, recall of 0.75, F1 score of 0.80, sensitivity of 0.75, specificity of 0.74, PPV of 0.84, and NPV of 0.62. The Clinical model showed generally lower values across most metrics in both cohorts, indicating that reliance on clinical factors alone limited predictive performance, whereas machine learning models substantially improved prediction.

**Table 3:** Comparison of the performance of the models.

Model	AUC	95%CI	ACC	PRE	REC	F1-score	SEN	SPE	PPV	NPV
Training cohort										
LR	0.92	0.88-0.97	0.81	0.91	0.79	0.84	0.79	0.85	0.91	0.68
SVM	0.85	0.78-0.91	0.76	0.85	0.75	0.80	0.75	0.76	0.85	0.63
KNN	0.88	0.83-0.94	0.79	0.78	0.94	0.85	0.94	0.50	0.78	0.82
XGBoost	0.88	0.82-0.94	0.79	0.87	0.79	0.83	0.79	0.78	0.87	0.67
Clinical	0.74	0.66-0.83	0.70	0.81	0.71	0.76	0.71	0.70	0.81	0.56
Combined	0.94	0.90-0.98	0.85	0.91	0.85	0.88	0.85	0.85	0.91	0.75
Validation cohort										
LR	0.85	0.78-0.92	0.76	0.84	0.77	0.80	0.77	0.74	0.84	0.63
SVM	0.81	0.73-0.89	0.76	0.84	0.77	0.80	0.77	0.74	0.84	0.63
KNN	0.73	0.64-0.82	0.69	0.81	0.67	0.74	0.67	0.72	0.81	0.54
XGBoost	0.82	0.74-0.89	0.75	0.84	0.75	0.80	0.75	0.74	0.84	0.62
Clinical	0.66	0.56-0.76	0.62	0.73	0.65	0.69	0.65	0.57	0.73	0.46
Combined	0.86	0.79-0.93	0.75	0.84	0.75	0.80	0.75	0.74	0.84	0.62

AUC, area under the curve; ACC, accuracy; CI, confidence interval; PRE, precision; SEN, sensitivity; SPE, specificity; REC, recall; PPV, positive predictive value; NPV, negative predictive value; LR, logistic regression; SVM, linear support vector machine; KNN, k-Nearest Neighbor; XGBoost, eXtreme Gradient Boosting. Fixed threshold = 0.5 for all metrics except AUC.

**Figure 4:**
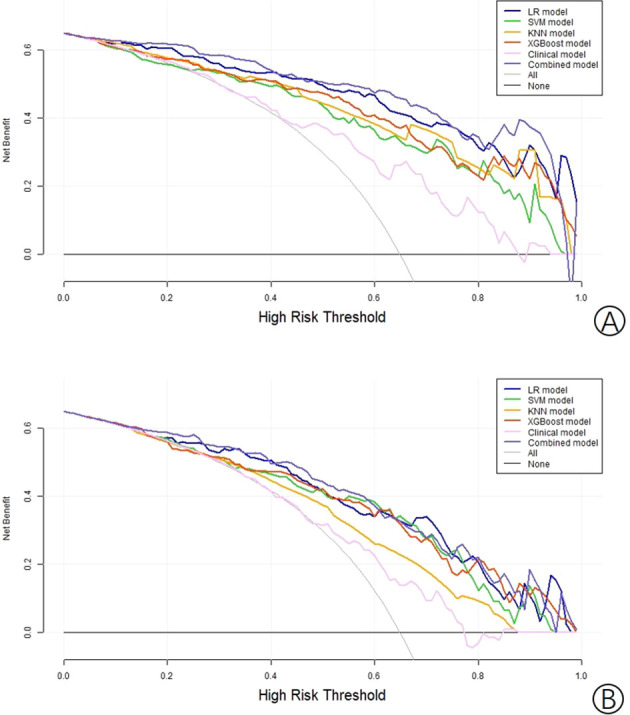
Decision curve analysis of the LR model, SVM model, KNN model, XGBoost model, clinical model, and combined model in the training cohort **(A)** and validation cohort **(B)**. The x-axes represent threshold probability, and the y-axes represent net benefit.

Calibration curves were used to assess the agreement between predicted probabilities and actual outcomes (**Supplementary Figure 2**). As seen in the figure, the calibrated curve closely approximated the ideal reference line, and the Hosmer-Lemeshow test yielded P values greater than 0.05 for all models. These findings indicate good calibration performance. Decision curve analysis was carried out to evaluate clinical net benefit and to assess model usefulness, as shown in **Figure 4**. Across a range of threshold probabilities (about 0.2 to 0.8), the combined model showed a higher net benefit than both the ‘treat all’ and ‘treat none’ strategies. It also outperformed the individual ML models and the clinical model alone. This suggests that using the combined model to guide early intervention may lead to better clinical outcomes than treating all patients or treating none. Treating all patients may expose many low-risk patients to unnecessary monitoring or treatment, while treating none may miss early detection of ICIP. The net benefit of the combined model was especially clear at threshold probabilities between 0.3 and 0.6, which are in line with practical decision thresholds for starting preventive measures or closer monitoring for ICIP.

## 3 Discussion

In the treatment of advanced lung cancer with ICIs, ICIP is a noteworthy adverse effect. Severe cases can be life-threatening and account for a substantial proportion of immunotherapy-related deaths. In a multicenter retrospective cohort study, the incidence of any-grade ICIP was reported as 12.0% among patients with NSCLC receiving pembrolizumab combined with chemotherapy, while the incidence of grade 3–5 ICIP was 4.1% (29). Similar incidence rates have been observed for anti-PD-1 or PD-L1 agents (30). When these agents are combined with anti-CTLA-4 drugs, such as ipilimumab, both the incidence and severity of ICIP may increase modestly (9). Among patients who develop grade 3–5 ICIP, the mortality rate can reach 10%–30% (31), particularly in cases with delayed diagnosis and treatment. Therefore, the establishment and implementation of an effective early prediction system have become a critical priority in clinical safety management.

The treatment of lung cancer has entered an era of precision medicine guided by biomarkers. Delta radiomics, compared with traditional radiomics, offers a core advantage by advancing from single time point static characterization to continuous and dynamic monitoring. This approach enables more precise capture of changes in tumor biological behavior under therapeutic intervention and addresses the inherent limitations of conventional methods (24, 25). Before initiating the present research, our team completed a series of systematic preliminary studies. These efforts not only validated the feasibility of the key technical pathway but also provided an essential parameter basis and optimization framework for the current study. First, we established baseline models, early treatment models, and delta models to predict progression-free survival (PFS) in patients with stage III–IV NSCLC using radiomic and delta radiomic features derived from CT images obtained before and early during ICIs treatment. The delta radiomic model achieved a C-index of 0.80 (95% CI: 0.75–0.85), which was superior to that of the baseline model with a C-index of 0.64 (95% CI: 0.57–0.71) and the early treatment model with a C-index of 0.75 (95% CI: 0.69–0.81) (P < 0.0001) (32). Subsequently, we analyzed delta radiomic features of primary tumors and metastatic lymph nodes in patients with stage III–IV NSCLC. We evaluated their predictive ability for treatment response to ICIs at 6 months, classifying patients as responders or non-responders. The nodal model based on delta radiomic features demonstrated excellent performance and outperformed the tumor model, with AUCs of 0.96 (95% CI: 0.90–1.00) and 0.86 (95% CI: 0.76–0.95), respectively (33). These findings highlight the strength of delta radiomics in early efficacy prediction and precise prognostic stratification. In the present study, delta radiomic features calculated from baseline and early treatment images enabled early and non-invasive risk stratification for ICIP in patients with advanced NSCLC receiving ICIs. This was achieved by quantifying dynamic changes in tumor texture heterogeneity, thereby providing valuable imaging-based biomarkers for ICIP risk identification and facilitating prospective clinical intervention. In 2017, Colen RR et al. (34) first explored the potential of radiomics for predicting ICIP risk, although only two positive cases were included. A subsequent study in 2022 (35) also had a relatively small sample size of 48 patients. In several recent studies (36, 37), investigators constructed traditional radiomics or deep learning models based on baseline images to predict ICIP risk. However, their predictive performance was lower than that of our delta radiomics model, with reported AUCs of 0.871 (95% CI: 0.786–0.956) and 0.865 (95% CI: 0.768–0.897), compared with an AUC of 0.92 (95% CI: 0.88–0.97) in the present study.

In this study, we systematically compared predictive models constructed using four machine learning algorithms, namely LR, SVM, KNN, and XGBoost. A five-fold cross-validation framework was applied to ensure fairness in performance comparison and robustness of the results. The overall findings indicated that the LR model achieved the highest predictive performance, whereas the KNN model performed relatively poorly. The SVM and XGBoost models demonstrated comparable predictive ability. These results have several implications. First, the superior performance of the LR model suggests a strong linear or approximately linear relationship between the delta radiomic features selected by LASSO and the risk of ICIP. As a parametric linear model, LR effectively captured the main trends and relationships in the data using a relatively simple decision boundary. A previous study (38) systematically compared multiple models for predicting heart failure readmission and mortality and found that traditional statistical models, such as LR and Cox proportional hazards models, performed comparably to or even slightly better than more complex machine learning models. This highlights the advantages of stability and interpretability of traditional models, particularly in settings with limited sample sizes. Second, the relatively weak performance of the KNN model is consistent with the intrinsic characteristics of this algorithm. As an instance-based lazy learning method, KNN relies heavily on distance metrics and is sensitive to irrelevant features and local noise in high-dimensional data (39). This sensitivity limits its robustness and generalization to unseen samples. Furthermore, although SVM and XGBoost achieved reasonable performance, both were slightly inferior to LR. While SVM can fit complex decision boundaries, especially with non-linear kernels, this flexibility may be unnecessary for data with predominantly linear relationships and may reduce generalization due to sensitivity to noise. XGBoost, as a powerful ensemble model, exhibited stable performance. However, its increased complexity did not translate into additional predictive gains in this study, indicating that non-linear relationships between features and outcomes were not dominant.

A large number of clinical studies have confirmed that patients with coexisting ILDs have a significantly higher risk of immune-related pneumonitis, particularly high-grade pneumonitis, after receiving immunotherapy compared with patients without ILDs. One study demonstrated that the presence of COPD [odds ratio (OR), 7.194; 95% CI: 1.130–45.798; P = 0.037] was independently associated with an increased incidence of ICIP. This finding confirms that pre-existing ILDs represent an independent risk factor for ICIP in patients with NSCLC and markedly elevate the likelihood of its occurrence (40). Patients with ILDs commonly exhibit chronic inflammation and fibrosis within lung tissue, accompanied by alveolar structural damage and impaired epithelial barrier function. This “vulnerable” pulmonary environment is more susceptible to excessive immune responses induced by immunotherapy. A low peripheral blood ALC prior to treatment has also been identified as an independent risk factor for the development of immune-related pneumonitis in lung cancer patients treated with ICIs. Although immunotherapy is designed to reactivate T cells to target tumors, this fragile immune background may predispose patients to uncontrolled inflammatory responses against normal lung tissue. Nishino M, et al. (41) reported in multiple analyses that a lower pre-treatment ALC is a significant risk factor for ICIP, including both any-grade and high-grade events. Compared with individual lymphocyte counts, the NLR provides a more comprehensive reflection of systemic inflammation and immune status and may offer more stable predictive value. An elevated NLR intuitively represents a dual imbalance characterized by enhanced “pro-inflammatory forces” and weakened “adaptive immune/regulatory forces.” This imbalance renders the body more vulnerable to excessive inflammation affecting normal tissues, such as the lung, following immune activation during immunotherapy, namely ICIP. Wang DY, et al. (42) conducted a recent meta-analysis that summarized evidence up to 2024 and clearly identified high NLR as a significant risk factor for ICIP. This analysis also provided pooled risk estimates, such as HR or OR, thereby quantitatively defining its impact.

In terms of model comparison, the LR model showed robust discriminative performance in both training (AUC 0.92) and validation sets (AUC 0.85). Adding clinical features (ILDs, ALC, NLR) to form a Combined model resulted in a numerically higher AUC in the validation set (0.86), but the improvement was not statistically significant (DeLong test p = 0.4691). Therefore, from a clinical implementation perspective, the simpler LR model—which requires only imaging data and no additional clinical variables—may be preferable due to its virtually equivalent validation performance and greater simplicity. The Combined model does not provide a statistically robust incremental benefit in this internal validation set.

A gap between training and validation performance was observed across all models. For the LR model, the AUC decreased from 0.92 in the training set to 0.85 in the validation set; the Combined model showed a similar decrease from 0.94 to 0.86. This may reflect some degree of residual overfitting. A contributing factor is the events-per-variable ratio: with 46 ICIP events and 22 retained features, the ratio was approximately 2.1. Although LASSO regularization and cross-validation were applied, a ratio below 5–10 may still allow some overfitting. Therefore, the validation set AUC (around 0.85) likely represents a more realistic estimate of model performance in future clinical practice. External validation in larger, independent cohorts remains necessary.

Although our correlation analysis did not reveal a systematic association between the magnitude of the top Delta-radiomics features and the TP0-TP1 interval, the timing of the follow-up CT still varied across patients (from after cycle 1 to cycle 3). For future clinical deployment, standardizing the timing of the follow-up CT — for example, consistently performing it after the second treatment cycle — would help minimize potential variability and improve model reproducibility. Prospective studies with predefined scan schedules are needed to confirm the robustness of the Delta-radiomics signature.

Most Delta-radiomics features were mainly texture-based (GLSZM and wavelet/LoG filters), which capture changes in tumor heterogeneity, edge sharpness, and local intensity distribution (43). Under ICIs, early lymphocytic infiltration and stromal remodeling around the tumor may disrupt regular tissue architecture, leading to measurable texture shifts. Peritumoral oedema and inflammatory cell accumulation — precursors of clinically detectable pneumonitis — could similarly alter wavelet-filtered features that represent multi-scale spatial patterns. Thus, the same immune activation that drives antitumor responses might also prime the lung for immune-related adverse events.

Our study has several limitations. First, this study was based on a single-center retrospective design and lacked external validation, which limits the evaluation of model generalizability. In addition, technical variability arising from differences in scanning devices and imaging protocols may influence the interpretation of radiomic features (44). External validation in independent cohorts, preferably multi-center cohorts, is needed before the model can be applied in clinical practice. Future studies should use prospective multi-center designs with standardized imaging protocols to further verify these findings. Second, although the LR model achieved the highest predictive accuracy and demonstrated notable advantages in deployment due to its simple structure, high computational efficiency, and strong interpretability, the dataset may reflect characteristics of specific populations or clinical environments. Future investigations could explore whether non-linear models, such as XGBoost, achieve improved performance after feature transformation, including the incorporation of interaction terms, in order to assess the limits of linear assumptions. Moreover, the inclusion of larger and more diverse datasets will be essential for evaluating the robustness and generalizability of the current optimal model. Third, the relatively small number of positive ICIP cases (N = 46) limited further analysis. It was not possible to carry out severity-based grading or stratified analysis based on key clinical factors. Among these cases, only a small number (n=8) were high-grade (grade 3–5) pneumonitis, while the remaining 38 were low-grade (grade 1–2) events. Therefore, all ICIP cases were analyzed as a single group. Future research should expand cohort size through multi-center collaboration to enable meaningful grading and stratification of ICIP, thereby elucidating the core factors that drive disease phenotype and severity and supporting the development of more precise risk prediction models. Fourth, the stability of the selected 22 radiomic features may be a concern. Given the relatively small sample size and high-dimensional feature space, the features selected by LASSO may be sensitive to small changes in the training data. Future multicenter studies with larger samples and standardized imaging protocols are needed to assess feature reproducibility and confirm the robustness of the model. Therefore, the proposed model should be considered a preliminary prediction tool rather than a clinically ready instrument.

In summary, this exploratory study provides preliminary evidence that Delta-radiomics features derived from early post-treatment CT images may help identify patients at higher risk of ICIP. The combined model shows promising discriminative performance in a single-center retrospective cohort. However, external validation in prospective, multi-center cohorts is essential before this model can be considered for clinical use. The current results should be viewed as hypothesis-generating and as a foundation for future research.

## Data Availability

The original contributions presented in the study are included in the article/**Supplementary Material**. Further inquiries can be directed to the corresponding author.
