# Supplementary material for: Early prediction of immune checkpoint inhibitor-related pneumonitis in advanced non-small cell lung cancer based on primary tumor delta-radiomics features
Source: Front Immunol. 2026 May 28;17:1795169. doi: 10.3389/fimmu.2026.1795169 (PMC13253751; doi:10.3389/fimmu.2026.1795169)
Supplement: Supplementary file 1 [file Table1.docx]

Supplementary Material

# Supplementary Tables

**Table S1.** List of the 22 selected Delta‑radiomics features with image filters, feature families, and LASSO coefficients.

| Feature Name | Filter | Feature Family | Coefficient |
| --- | --- | --- | --- |
| square_gldm_DependenceVariance | square | GLDM | -0.0322 |
| exponential_glcm_InverseVariance | exponential | GLCM | 0.1225 |
| wavelet-HLH_glszm_SmallAreaEmphasis | wavelet-HLH | GLSZM | 0.1203 |
| exponential_firstorder_Skewness | exponential | First-order | -0.0720 |
| squareroot_firstorder_10Percentile | squareroot | First-order | 0.0660 |
| log-sigma-5-0-mm-3D_glszm_HighGrayLevelZoneEmphasis | log-sigma-5-0-mm-3D | GLSZM | 0.0897 |
| square_glcm_ClusterShade | square | GLCM | -0.0052 |
| wavelet-LLH_glrlm_LongRunLowGrayLevelEmphasis | wavelet-LLH | GLRLM | -0.0296 |
| wavelet-LLH_glszm_SmallAreaEmphasis | wavelet-LLH | GLSZM | 0.0756 |
| logarithm_glrlm_ShortRunLowGrayLevelEmphasis | logarithm | GLRLM | -0.0667 |
| wavelet-HHL_firstorder_Skewness | wavelet-HHL | First-order | -0.0178 |
| log-sigma-5-0-mm-3D_glrlm_LowGrayLevelRunEmphasis | log-sigma-5-0-mm-3D | GLRLM | -0.0631 |
| log-sigma-1-0-mm-3D_glszm_SmallAreaLowGrayLevelEmphasis | log-sigma-1-0-mm-3D | GLSZM | 0.0124 |
| logarithm_glszm_SmallAreaHighGrayLevelEmphasis | logarithm | GLSZM | 0.0132 |
| logarithm_ngtdm_Strength | logarithm | NGTDM | 0.0469 |
| wavelet-HHH_glrlm_HighGrayLevelRunEmphasis | wavelet-HHH | GLRLM | -0.0374 |
| wavelet-HLL_glcm_Imc2 | wavelet-HLL | GLCM | -0.0113 |
| wavelet-HHH_glszm_ZoneEntropy | wavelet-HHH | GLSZM | -0.0449 |
| log-sigma-5-0-mm-3D_glszm_LargeAreaLowGrayLevelEmphasis | log-sigma-5-0-mm-3D | GLSZM | -0.0170 |
| wavelet-HLL_glszm_LargeAreaLowGrayLevelEmphasis | wavelet-HLL | GLSZM | 0.0016 |
| exponential_glszm_SmallAreaEmphasis | exponential | GLSZM | 0.0008 |
| log-sigma-5-0-mm-3D_glszm_SmallAreaEmphasis | log-sigma-5-0-mm-3D | GLSZM | -0.0040 |

GLCM, Gray Level Co-occurrence Matrix; GLDM, Gray Level Dependence Matrix; GLRLM, Gray Level Run Length Matrix; GLSZM, Gray Level Size Zone Matrix; LoG, Laplacian of Gaussian; NGTDM, Neighborhood Gray Tone Difference Matrix

**Table S2.** Spearman correlations between top 5 Delta‑radiomics features (by absolute LASSO coefficient) and the TP0‑TP1 interval.

| Feature | LASSO coefficient | Spearman ρ | P value |
| --- | --- | --- | --- |
| exponential_glcm_InverseVariance | 0.1225 | -0.133 | 0.130 |
| wavelet‑HLH_glszm_SmallAreaEmphasis | 0.1203 | 0.087 | 0.326 |
| log‑sigma‑5‑0‑mm‑3D_glszm_HighGrayLevelZoneEmphasis | 0.0897 | 0.052 | 0.558 |
| wavelet‑LLH_glszm_SmallAreaEmphasis | 0.0756 | -0.041 | 0.641 |
| exponential_firstorder_Skewness | 0.0720 | 0.001 | 0.988 |

# Supplementary Figures


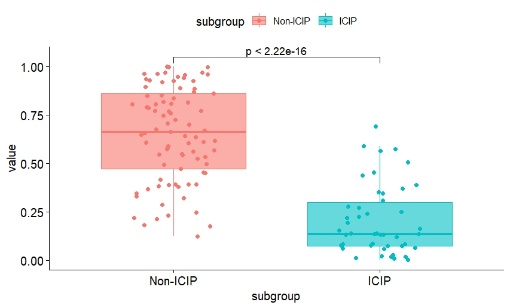

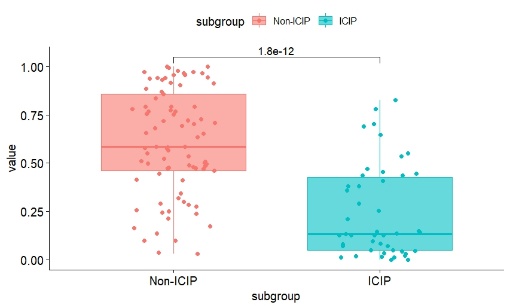


A B


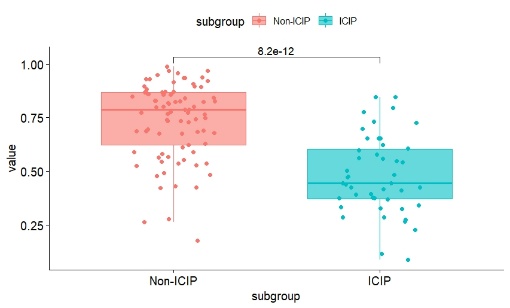

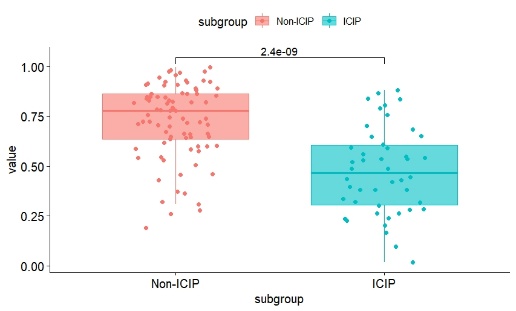


C D


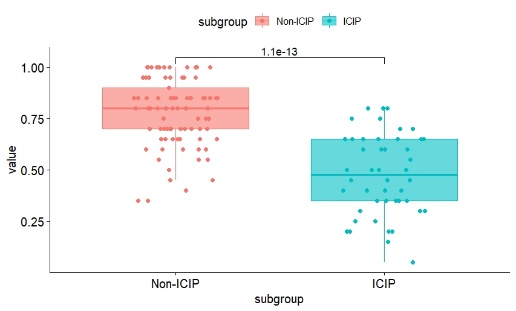

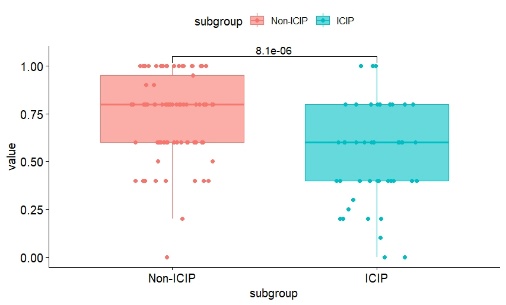


E F


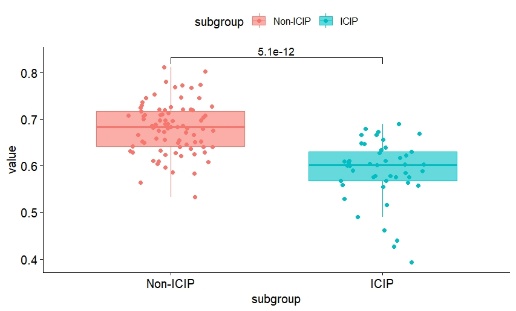

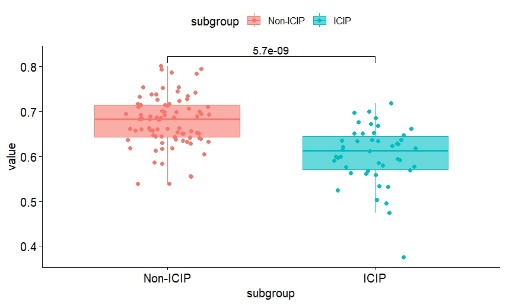


G H


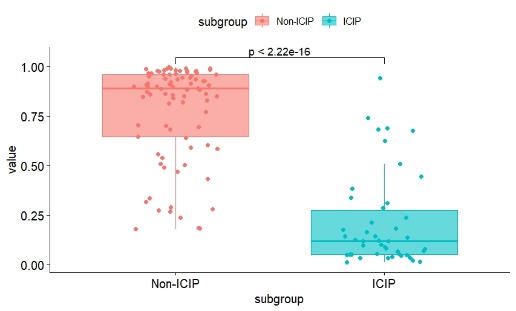

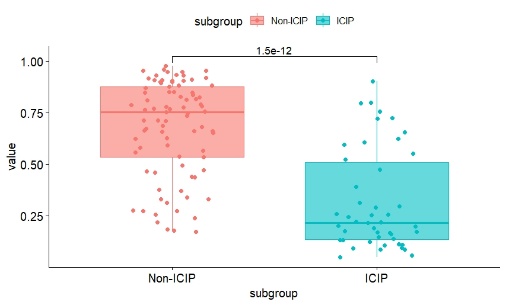


I J

**Figure S1.** Significant differences in the distribution of delta radiomics scores were observed between the ICIP and Non-ICIP groups. Training set: LR model(A), SVM model(C), KNN model(E), XGBoost model(G), Combined model(I); Validation set: LR model(B), SVM model(D), KNN model(F), XGBoost model(H), Combined model(J).


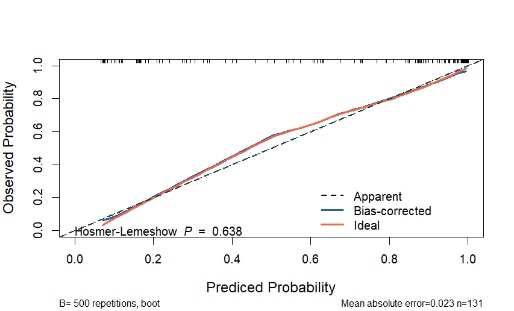

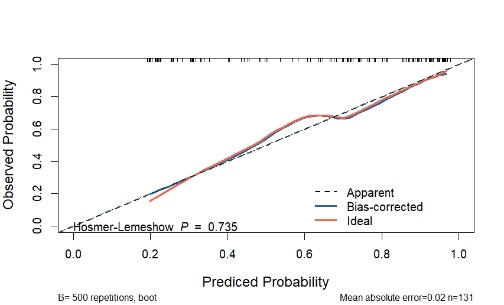


A B


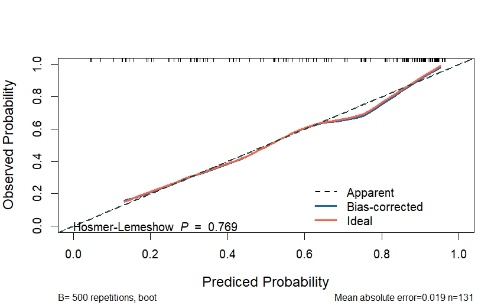

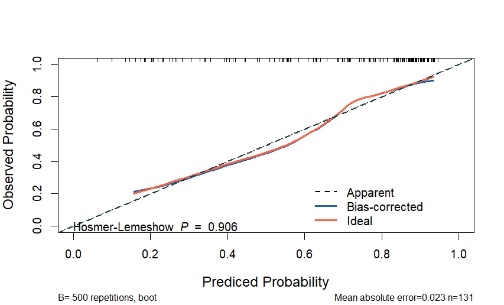


C D


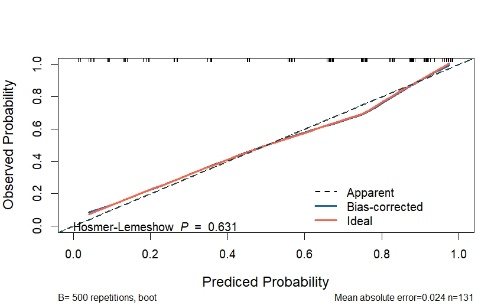

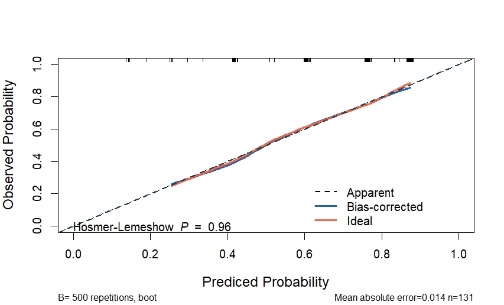


E F


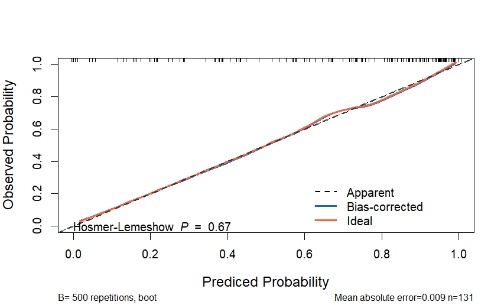

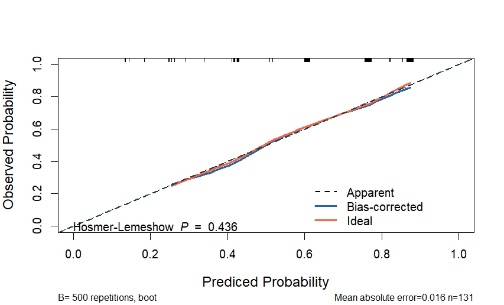


G H


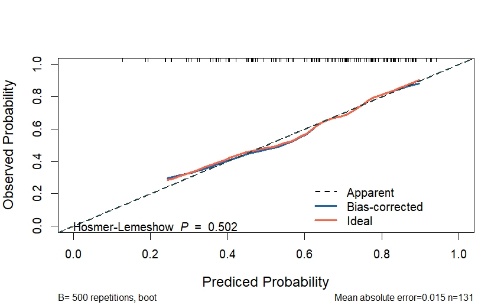

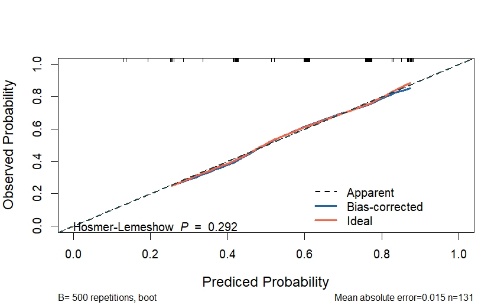


I J


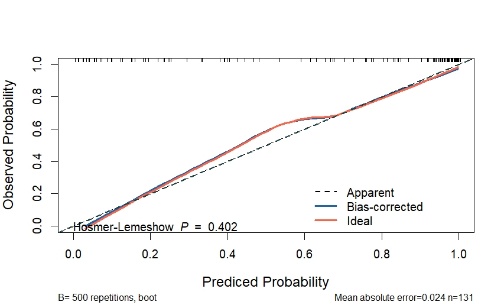

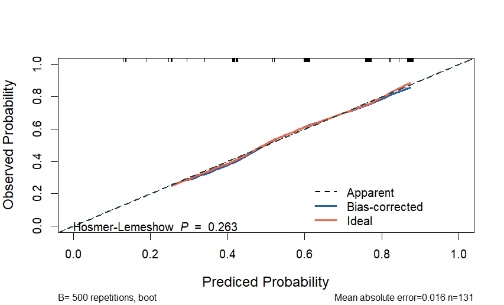


K L

**Figure S2.** Calibration curves were used to assess the agreement between predicted probabilities and actual outcomes(P＞0.05). Training set: LR model(A), SVM model(C), KNN model(E), XGBoost model(G), Clinical model(I), Combined model(K); Validation set: LR model(B), SVM model(D), KNN model(F), XGBoost model(H), Clinical model(J), Combined model(L).
